# Emergency Department Visits Before, After and During Integrated Home Care: A Time Series Analyses in Italy

**DOI:** 10.34172/ijhpm.2022.6662

**Published:** 2022-05-29

**Authors:** Sara Campagna, Alessio Conti, Valerio Dimonte, Paola Berchialla, Alberto Borraccino, Maria Michela Gianino

**Affiliations:** ^1^Department of Public Health and Pediatrics, University of Torino, Torino, Italy.; ^2^Department of Clinical and Biological Sciences, University of Torino, Torino, Italy.

**Keywords:** Home Care Services, ED Use, Retrospective Study, Sevice Evaluation, Electronic Medical Records

## Abstract

**Background:** Integrated home care (IHC) is one strategy to provide care to people with multiple chronic conditions, and it contributes to the reduction of unnecessary emergency department (ED) use, but there are conflicting results on its effectiveness. In this study, we assessed the frequency and characteristics of ED visits occurring before, during, and after IHC in a large cohort of IHC patients enrolled over 6 years.

**Methods:** The analysis included 39 822 IHC patients identified in Italian administrative databases. Patients were grouped in tertiles according to IHC duration (short, intermediate, and long) and the number of ED visits during IHC was compared to that the 12 months before IHC enrolment and in the 12 months after IHC discharge across IHC duration groups.

**Results:** We observed a reduction in ED visits during IHC. IHC was significantly associated with a reduction in ED visits in the long and short IHC duration groups. A non-significant reduction in ED visits was observed in the intermediate IHC duration group. A 90% reduction in ED visits during IHC and a 45% reduction after IHC was observed in the short IHC duration group. Corresponding reductions were 17% and 64% during and after IHC, respectively, in the long IHC duration group.

**Conclusion:** IHC was effective in reducing ED visits, but expansion of IHC to include additional necessary services could further reduce ED visits. Investment in the creation of a structured, effective network of engaged professionals (including community care services and hospitals) is crucial to achieving this.

## Background

**Key Messages**

** Implications for policy makers**

- Integrated home care (IHC) significantly decreased emergency department (ED) visits, in the short and long IHC duration groups.
- IHC showed to have a positive impact in reducing inappropriate ED visits, the severity of ED triage codes registered increased during IHC in all groups.
- To better answer population needs, IHC should be further improved by increasing the number of dedicated staff, operating hours, and overall availability.
- A structured network including community care services and hospitals may further help in limiting ED use for deferable conditions.

** Implications for the public**

The integrated home care (IHC) system provides care to people with multiple chronic conditions impacting their quality of life. Our study showed that during IHC, patients had a reduced number of emergency department (ED) visits, and it was independent of IHC duration. The overall results suggested that the IHC is a suitable strategy to respond to specific population needs, and would further benefit from improving the number and availability of dedicated staff, in particular by increasing the operating hours. To reduce inappropriate ED access occurrence, the IHC system could advantage from the establishment of specific care coordinated pathways that would simplify access to necessary services which, at the state, are currently unavailable or impractical to perform in patients’ homes. Investment in the creation of a structured, effective network of engaged professionals (including community care services and hospitals) could be the driver to promote the creation of new care pathways and interprofessional collaboration in the IHC system, thus further limiting ED visits.

Providing care to adults with multiple chronic conditions is a major challenge for healthcare systems, because these individuals require more services as their age, frailty, and dependence increase.^1^ Many patients with disabilities related to chronic conditions and comorbidities are homebound, and when their condition is coupled with social frailty (ie, isolation, poverty), these patients become even more vulnerable. Home care services offer support in activities of daily living and in the management of complex medical conditions, while keeping patients in a familiar setting. In many European countries, enhanced community services like home care are considered a potentially effective way for people to maintain their independence; these services are also the preferred choice of most patients.^2^

 The Italian National Healthcare System is publicly funded and began offering home care services to all citizens in 2001. The aim of these services is to provide continuous, integrated health and social assistance delivered by family practitioners and other professionals (eg, nurses, social workers, therapists, and medical specialists as appropriate)^3^ at the patient’s home. Home care services are implemented based on regional regulations, and two models are available depending on the intensity, complexity, and duration of the care intervention. The first is programmed home care, in which family practitioners or nurses care for patients at their home, performing clinical consultations or simple technical procedures on a set schedule (weekly or monthly), and family practitioners programme hospitals visits as needed. The second model is integrated home care (IHC), which provides different professional services in response to medium-/high-complexity medical, nursing, and/or rehabilitation and social health needs,^4^ such as post-surgery discharge, acute medical conditions, or long-term care and rehabilitation support. Patients can be enrolled in IHC upon specific request from a citizen (eg, a family member), a family practitioner, or a hospital. Local Geriatric Assessment Units are responsible for choosing the appropriate home care service model based on the patient’s health and social needs. IHC is coordinated by the patient’s family practitioner, and it is delivered by an IHC team between 8:00 am to 8:00 pm on week days. An active on-call medical service delivers the service between 8:00 pm to 8:00 am on week days, and is available 24 hours a day during week-ends.^5^ The overall aim of home care is to improve patients’ quality of life, psychological and physical well-being and that of their family, and reduce the costs associated with long and/or repeated hospitalisations. In addition, IHC is used to provide appropriate palliative care to terminally ill patients. IHC models like the one in Italy are assumed to facilitate hospital discharge and have been shown to ensure continuity of care, avoid unnecessary hospital visits or readmission, and reduce overall costs and emergency department (ED) visits.^6^ Moreover, recent studies have shown that patients who cannot benefit from effective home care services tend to have more unplanned ED visits, unnecessary hospitalisations, and nursing home placements.^7,8^

 Although promising, results vary on the effectiveness of home care services to reduce ED visits.^9-14^ For example, Jones et al reported that ED visits increased following same-day nursing home care visits.^11^ However, to the best of our knowledge, there are no large, long-term, population-based studies comparing ED visits before, during, and after IHC. Understanding the real-life patterns and characteristics of ED visits among people who have received IHC may help policymakers determine if such services need qualitative and/or quantitative improvements. Hence, we assessed the frequency and characteristics of ED visits occurring before, during, and after IHC in a large cohort of IHC patients enrolled over 6 years.

## Methods

###  Design

 This retrospective observational study was conducted in the second largest region of Italy, the Piedmont Region, which has an area of 25 387 km^2^ and a population of about 4.4 million inhabitants.^15^

###  Sample

**Figure 1:**
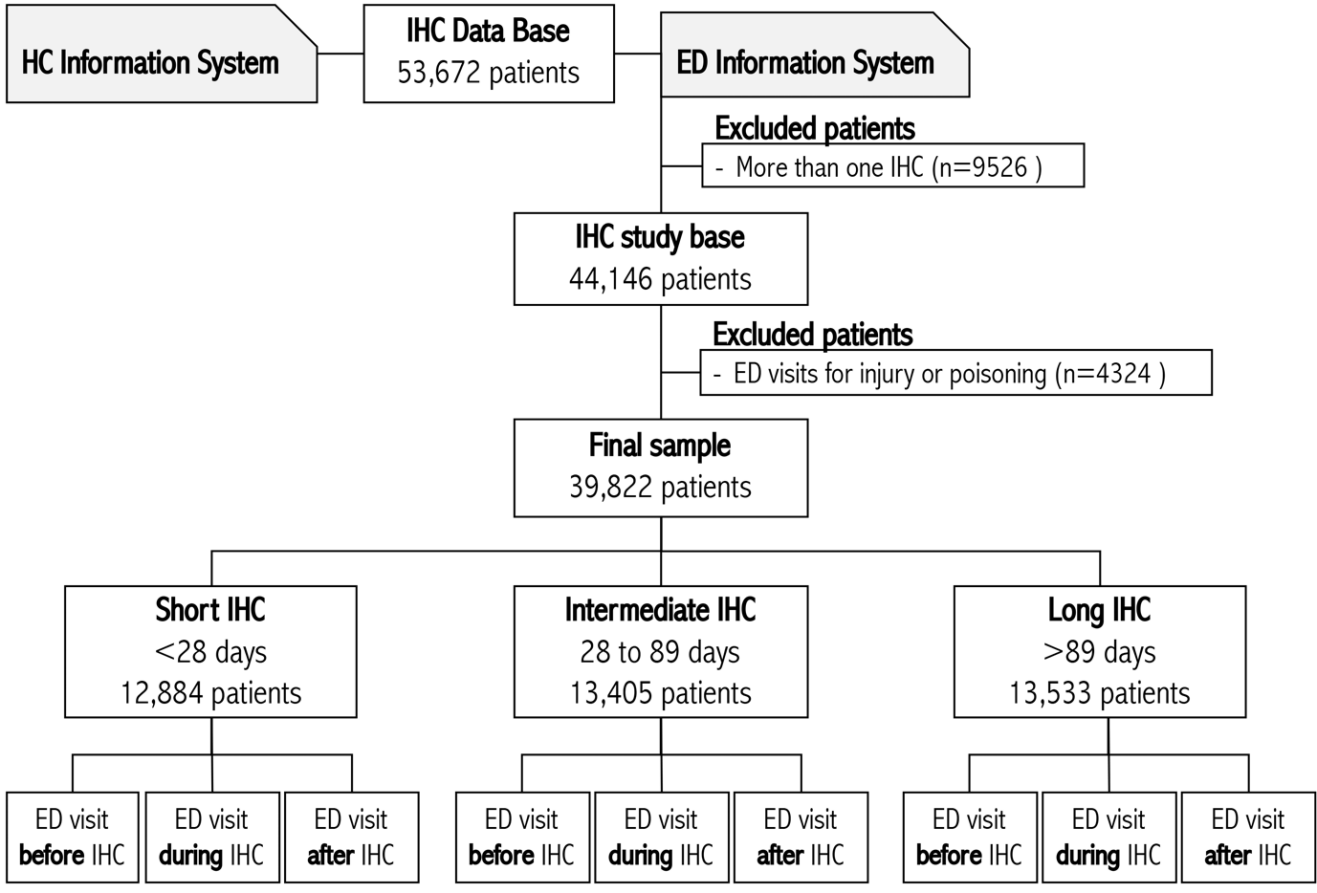
Study flowchart: Piedmont, Italy, 2012-2017. Abbreviations: HC, home care; ED, emergency department; IHC, integrated home care.

 We included a population-based cohort of patients who were enrolled in IHC between January 1, 2012 and December 31, 2017, and were alive 12 months after discharge from IHC. Patients were identified from administrative, ministerial, compulsory electronic medical records. Further details on the databases used and the sampling procedure can be found in the parent study.^14^ Patients enrolled in home care models other than IHC (eg, programmed home care or palliative home care) were excluded. If a patient had more than one IHC episode during the enrolment period, only the first episode was considered. ED visits with a registered International Classification of Diseases (ICD) diagnostic code of “injury and poisoning” were also excluded, as such visits are unlikely to be related to the chronic conditions that provoked the patient’s enrolment in IHC. Therefore, the final sample consisted of 39 822 patients (Figure 1).

###  Data Collection

 Data were collected from two databases: the Home Care Information System “SIAD (*Sistema Informativo Assistenza Domiciliare*)” database, which is the official Italian National Information Monitoring System for Home Care Services, and the Italian National Information System for ED use database. Patients are registered in these databases using a unique assigned identification number, which makes it possible for researchers to perform data linkage while protecting anonymity. Since these databases are open to authorised academic and research institutions for epidemiological studies, ethical committee approval was not sought.

 We extracted the following information on patient characteristics and on ED visits that occurred 12 months before IHC enrolment, during IHC, and 12 months after IHC discharge, from the aforementioned databases: gender, age, duration of IHC, prevalent disorder at IHC enrolment, ED triage code (white – non urgent; green – less urgent; yellow – urgent; red – immediate), ED triage symptoms, diagnosis registered at ED visit (ICD-9 code), time of arrival at ED (8:00 am-4:00 pm, 4:00 pm-12:00 pm, 12:00 pm-8:00 am), and destination after discharge from ED (hospital, home, other facility). Patients were categorised by tertile of IHC duration: short (1-27 days), intermediate (28-89 days), and long (90-2184 days), and the number of ED visits during IHC were compared to the number in the 12 months before IHC enrolment and in the 12 months after IHC discharge, across IHC duration groups.

###  Analysis

 Continuous variables were described as median and interquartile range; categorical variables were described as frequency and percentage. Characteristics of ED visits were described according to their timing (before, during, and after IHC) and stratified by IHC duration (short, intermediate, and long) to account for different health statuses. An interrupted time series analysis was adopted using a Type II Sum Squares ANCOVA (analysis of covariance) Lagged Dependent Variable model to estimate the average differences in the number of ED visits before and during IHC, and after and during IHC.^16^ We chose this analysis because we collected the monthly number of ED visits, and the interrupted time series analysis allows the time series of ED visits before IHC to be used to provide a counterfactual for what would have occurred in the absence of IHC. The model parameters were estimated by bootstrap resampling. Average differences in the number of ED visits before and during IHC, and after and during IHC, are reported. The Type II Sum Squares ANCOVA Lagged Dependent Variable model was chosen based on the limited number of observations. The significance level was set at 0.05. All analyses were carried out using software R version 3.6.1.^17^

## Results

 We observed similar male-to-female ratio across the short, intermediate, and long IHC duration groups, although female patients were significantly more represented in the long IHC duration group. Median age in the whole cohort of 39 822 patients was 80 years. The most prevalent disorders at IHC enrolment were cardiovascular diseases and cancer (Table).

**Table:** Patient Characteristics, IHC Duration, and Prevalent Disorder at IHC Enrolment (Piedmont Region, Italy, 2012-2017)

	**All IHC**	**IHC Duration**			***P*** ** Value**
		**<28 Days**	**From 28 to 89 Days**	**>89 Days**	
Patient's age (years), Median (IQR)	80 (69-87)	81 (71-88)	80 (70-87)	79 (68-86)	<.001
IHC duration (days), Median (IQR)	51 (19-120)	12 (6-19)	50 (37-66)	173 (118-278)	<.001
Gender, No. (%)					
Females	20896 (52.5)	6421 (49.8)	7168 (53.5)	7307 (54.0)	<.001
Prevalent disorder at IHC enrolment, No. (%)					<.001
Neoplasms	12193 (30.8)	4819 (37.7)	3829 (28.7)	3545 (26.3)	
Cardiovascular diseases	5678 (14.3)	1677 (13.1)	1863 (14.0)	2138 (15.9)	
Trauma and injury	4222 (10.7)	548 (4.3)	1825 (13.7)	1849 (13.7)	
Neurological disorders	2913 (7.4)	2720 (5.6)	966 (7.2)	1227 (9.1)	
Endocrine and metabolic diseases	2248 (5.7)	824 (6.4)	762 (5.7)	662 (4.9)	
Mental disorders	1833 (4.6)	580 (4.5)	624 (4.7)	629 (4.7)	
Respiratory diseases	1729 (4.4)	816 (6.4)	486 (3.6)	427 (3.2)	
Skin diseases	1676 (4.2)	443 (3.5)	525 (3.9)	708 (5.3)	
Musculoskeletal diseases	1580 (4.0)	295 (2.3)	661 (5.0)	624 (4.6)	
Other	5527 (14.0)	2054 (16.1)	1800 (13.5)	1673 (12.4)	
All Patients, No. (%)	39822 (100.0)	12884 (32.3)	13405 (33.7)	13533 (34.0)	

Abbreviations: IHC, integrated home care; IQR, Interquartile range.

 Characteristics of ED visits occurring before, during, and after IHC are reported for all IHC duration groups in the supplementary material. We observed a significant drop in non-urgent and less urgent ED triage codes during IHC in all IHC duration groups, although these still represented 50.8%, 58.7%, and 61.0% of all ED triage codes for the short, intermediate, and long IHC duration groups, respectively (Supplementary file 1). We noticed a decrease in respiratory ED triage symptoms from before to during IHC in all IHC duration groups, an excess in symptoms of fever in the intermediate IHC duration group, and of uro-gynaecological symptoms and fever in the long IHC duration group. In the same time frame, the short and intermediate IHC duration groups had fewer ED visits between 8:00 am and 4:00 pm, and between 12:00 am and 8:00AM, but had increased ED visits between 4:00 pm and 12:00 am. More than 70% of all ED visits occurred between 8:00 am and 8:00 pm. Discharge to home accounted for 36.8% to 55.7% of ED visits across IHC duration groups.

**Figure 2:**
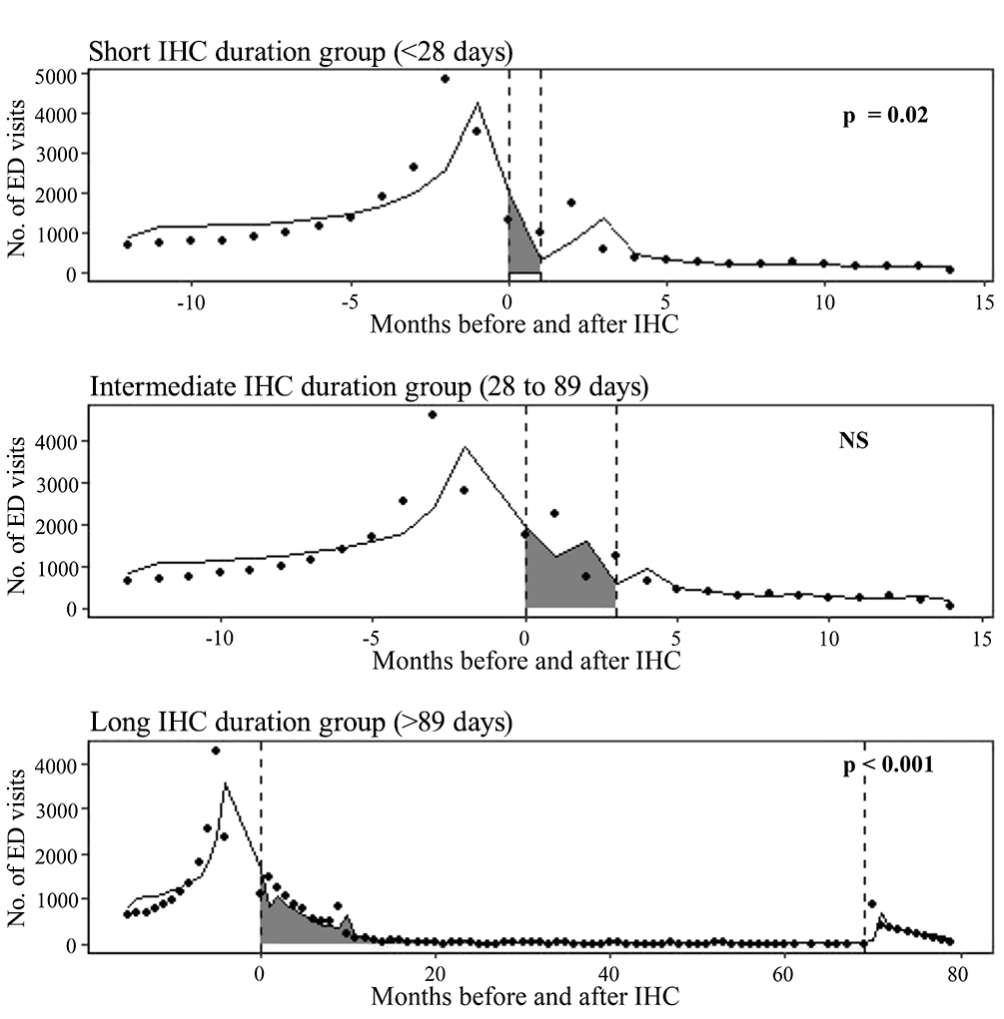
Number of ED Visits in the 12 Months Before IHC Enrolment and in the 12 Months After IHC Discharge by IHC Duration Group. Grey area represents IHC duration. *P* value for the difference in the number of ED visits before/during and during/after IHC. Piedmont Region, 2012-2017, Italy. Abbreviations: ED, emergency department; IHC, integrated home care; NS, not significant.

 The interrupted-time series analyses gave rates of ED visits before, during, and after IHC. Figure 2 reports the changes in level and slope in the 12 months after IHC discharge compared to the 12 months before IHC enrolment. In other words, it showed the immediate (level) changes in the rate of ED visits as well as changes in the trend. The ANCOVA Lagged Dependent Variable model showed that IHC was generally associated with a reduction in ED visits, although that observation was significant in the long and short IHC duration groups only. We observed a 90% reduction in ED visits during IHC and a 45% reduction after IHC in the short IHC duration group; corresponding reductions were 17% and 64%, respectively, in the long IHC duration group.

## Discussion

 In the context of healthcare system assessment, hospital readmissions and ED use for non-urgent care are usually considered indicators of sub-optimal healthcare in the primary setting.^18^ This study aimed to assess associations between IHC and unplanned ED visits in a large cohort of adults.

 Our study showed significant decreases in ED visits during and after IHC in the short and long IHC duration groups. Similar results have been reported in other contexts with smaller samples, and only after specific interventions delivered by geriatric units,^19^ multidisciplinary teams,^19^ or nurse practitioners.^20^ Similar findings have also been reported by a study examining differences in ED visits among long-term community care services and nursing facilities.^20^

 The reduction in ED visits we observed could mean that IHC provides an adequate support system for patients discharged from the hospital, even if they are clinically frail or live in socially frail conditions. Indeed, recent data from over 400 000 ED visits showed that receiving home care decreased the risk of ED visits in older individuals living in socially deprived areas (aged 65-84 years).^21^ Thus, IHC could promote efficient healthcare system utilisation not only due to its clinical aspects, but also by mitigating the effects of living in deprived areas.

 In our sample, the consistent reduction in ED visits was associated with a proportional increase in the severity of ED triage codes in all IHC duration groups, with more urgent and immediate ED codes observed during IHC and in the 12 months after IHC discharge. In addition, the number of patients discharged from ED to hospital or other facilities increased significantly compared to the number discharged to their home during IHC for all IHC duration groups. This result can be interpreted as a positive aspect of the IHC service, as patients reduced overall ED visits. On the other hand, it is possible that the concurrent increase in the severity of patients accessing to ED is due to a delay in expert care or specialist consultation. Unfortunately, this cannot be determined due to the nature of our data, which is a limitation of the current study. However, we can hypothesise that the ED visits that occurred during IHC were unavoidable, and that the conditions that provoked these visits required a higher level of care and thus could not have been treated at home, which is coherent with previous literature.^22^

 More than a third of all ED visits in our sample resulted in a discharge to the patient’s home, suggesting that, in some cases, the IHC team may have used an ED visit for diagnostic purposes, such as to conduct immediate imaging and/or invasive procedures. This seems especially plausible when only after-hours or on-call services are available, when the necessary procedures cannot be performed at the patient’s home,^23^ or after a home care nursing visit reveals deterioration that deserves deeper clinical evaluation.^11^ Indeed, in our sample about 6% of ED visits were used for routine healthcare services. This finding supports previous results highlighting the need to design a specific pathway by which people in IHC can access routine services in order to avoid inappropriate ED visits.^24^ In the last years, the Italian Government’s focus, beyond strengthening IHCs and creating a structured network, has been on implementing telemedicine to enable physicians to provide remote monitoring, diagnosis, and consultations.^25^

 Coherently with previous studies,^26-28^ respiratory disorders were the most frequent ED triage symptoms. Furthermore, about a quarter of ED visits in our sample did not result in a formal diagnosis. As shown in a previous work, patients usually visit the ED for baseline clinical conditions^14^. Due to the lack of diagnostic instrumentation and communication issues while in home care, IHC teams cannot determine the possible risk of mild or undefined symptoms, and their ability to identify and manage more critical situations is limited. Lastly, ED triage codes and symptoms may not have captured the real cause of ED visits among IHC patients; they may not be appropriate for the identification of disease exacerbation or the need for urgent treatment such as blood tests and instrumental exams.

 Nurses and nurse aides in Italy can easily provide low-complexity interventions like blood sample collection, continence care (including the management of bladder catheters), and personal hygiene to IHC patients; whereas diagnostic procedures of a higher complexity, like therapeutic treatments (eg, hemotransfusion and dialysis), rehabilitation treatments, and respiratory re-education, require a general practitioner or specialist intervention. As these healthcare professionals are not continuously available, these services cannot always be guaranteed to IHC patients.^29^ This may result in increased unplanned hospital or ED visits. Another aspect that may influence ED visits is the limited working hours of home care services, which consist mostly of daytime hours on weekdays.

 Recent Italian regulations^25^ allocated specific financial resources to expand IHC services. At present, about 3% of patients in Italy are receiving IHC,^30^ and this number is expected to increase to 10% by 2026, specifically for the elderly, people with chronic illnesses, and those with disabilities. Moreover, DL34/2020, which was released by the Italian Government during the coronavirus disease 2019 (COVID-19) pandemic, also called for the recruitment of 9600 family and community nurses to reach a ratio of 8 nurses for every 50 000 inhabitants.^31^ These nurses will be allocated within a network of community outpatient multidisciplinary care services and nurse-led community hospitals. These services and the care they deliver will be coordinated by new, territory-based operation centres. These changes could increase the availability and potential effectiveness of IHC and, consequently, reduce ED visits by creating an effective network aimed at supporting IHC teams in the diagnosis and treatment of complex situations and guarantee fast-track access to hospitals or other primary care facilities. Promoting around-the-clock availability of healthcare professionals could further help ensure prompt interventions, 24 hours a day, 7 days a week.

 These findings should be considered in light of the study’s limitations. Major limitations are those linked to the sources of information, which are common to all studies based on administrative databases. These limitations include problems related to the quality of data, including low accuracy in data collection and different coding criteria adopted across the institutions and individuals charged with populating these databases. Moreover, clinical and socio-demographic information, frailty and complexity index scores, and details on the level of care delivered are often not recorded. Finally, data on possible scheduled hospital use were not available. In contrast, administrative databases represent the best available sources for large-scale epidemiological studies and for monitoring population trends in service utilisation. It should be further noted that the lack of a control group also constitutes a relevant limitation of the current study.

 Among the strengths of the present study are the fact that it deals with an issue that is common to all public healthcare systems, ie, that they have been called to increase IHC availability and effectiveness; the databases used allowed us to investigate a large number of cases; and subgroup analyses by duration of IHC could represent a proxy for patient complexity.^32^ Finally, as the study was based in a large northern Italian region that has healthcare systems and population health profiles comparable to those of other European countries, findings could be generalised to other universal, publicly-funded healthcare systems.

## Conclusion

 IHC was effective in reducing ED visits. Improvements to the IHC system in Italy in terms of population coverage, organisation, skill-mix, and daily availability are planned, but implementation of these improvements at the national and local level will be a challenge. If successful, they will lead to further reductions in ED visits. Healthcare systems could benefit from the establishment of specific care pathways and coordination of care, to simplify access to necessary services that are not currently available nor practical to perform in patients’ homes. Investment in the creation of a structured, effective network of engaged professionals (including community care services and hospitals) is crucial to IHC. The implementation of this new organisational model requires a cultural shift that could be encouraged by specific education programmes that promote interprofessional collaborations and strengthen the network.

## Ethical issues

 Anonymised administrative database was used and managed according to the current deontological rules for the processing of National Statistical System data, therfore committee approval was not required.

## Competing interests

 Authors declare that they have no competing interests.

## Authors’ contributions

 SC: conceptualization, data curation, methodology, project administration, writing of the original draft, writing–review and editing. AC: data curation, methodology, writing original draft, writing–review and editing. VD: data curation, methodology, writing of the original draft, writing–review and editing. PB: conceptualization, data curation, formal analysis, methodology, writing of the original draft, writing–review and editing. AB: conceptualization, data curation, formal analysis, investigation, methodology, project administration, writing of the original draft, writing–review and editing. MMG: conceptualization, data curation, formal analysis, investigation, methodology, project administration, writing of the original draft, writing–review and editing.

## Supplementary files

Supplementary file 1 contains Table S1.
