# Supplementary material for: Emergency Department Visits Before, After and During Integrated Home Care: A Time Series Analyses in Italy
Source: Int J Health Policy Manag. 2022 May 29;11(12):3012–8. doi: 10.34172/ijhpm.2022.6662 (PMC10105177; doi:10.34172/ijhpm.2022.6662)
Supplement: Supplementary file 1 — contains Table S1. [file ijhpm-11-3012-s001.pdf]

**Article title:** Emergency Department Visits Before, After and During Integrated Home Care: A Time Series Analyses in Italy

**Journal name:** International Journal of Health Policy and Management (IJHPM)

**Authors' information:** Sara Campagna[1], Alessio Conti[1], Valerio Dimonte[1], Paola Berchialla[2], Alberto Borraccino[1]*, Maria Michela Gianino[1]

[1]Department of Public Health and Pediatrics, University of Torino, Torino, Italy.

[2]Department of Clinical and Biological Sciences, University of Torino, Torino, Italy

(*Corresponding author: Email: alberto.borraccino@unito.it)

## Supplementary file 1

**Table S1.** ED visits characteristics before, during and after IHC, stratified by short, intermediate and long IHC duration. Piedmont Region 2012-2017, Italy

| | **SHORT IHC DURATION (n=27 417)** | | | | **INTERMEDIATE IHC DURATION (n=28 818)** | | | | **LONG IHC DURATION (n=31 709)** | | | |
|---|---|---|---|---|---|---|---|---|---|---|---|---|
| | **Before IHC** | **During IHC** | **After IHC** | | **Before IHC** | **During IHC** | **After IHC** | | **Before IHC** | **During IHC** | **After IHC** | |
| | N (%) | N (%) | N (%) | **p-value** | N (%) | N (%) | N (%) | **p-value** | N (%) | N (%) | N (%) | **p-value** |
| **Accesses** Number | 20 625 | 2292 | 4500 | | 19 362 | 4779 | 4677 | | 18.417 | 10.438 | 2.854 | |
| **Accesses** Mean | 2.1 | 1.1 | 1.5 | | 2.0 | 1.3 | 1.5 | | 2.0 | 1.8 | 1.4 | |
| **ED TRIAGE CODE** | | | | | | | | | | | | |
| White (Non-urgent)[a] | 755 (3.7) | 27 (1.2) | 139 (3.1) | | 927 (4.8) | 93 (1.9) | 182 (3.9) | | 790 (4.3) | 228 (2.2) | 111 (3.9) | |
| Green (Less urgent) | 12 980 (62.9) | 1136 (49.6) | 2374 (52.8) | <0.001 | 12 028 (62.1) | 2714 (56.8) | 2534 (54.1) | <0.001 | 11 369 (61.7) | 6136 (58.8) | 1505 (52.7) | <0.001 |
| Yellow (Urgent) | 6383 (30.9) | 921 (40.1) | 1645 (36.6) | | 5879 (30.4) | 1696 (35.5) | 1658 (35.5) | | 5641 (30.6) | 3599 (34.5) | 1031 (36.1) | |
| Red (Immediate) | 507 (2.5) | 208 (9.1) | 342 (7.6) | | 528 (2.7) | 276 (5.8) | 303 (6.5) | | 617 (3.4) | 475 (4.5) | 207 (7.3) | |
| **ED TRIAGE SYMPTOMS** | | | | | | | | | | | | |
| Respiratory | 2169 (10.5) | 401 (17.5) | 691 (15.4) | | 1831 (9.5) | 680 (14.2) | 644 (13.8) | | 1740 (9.5) | 1378 (13.2) | 460 (16.1) | |
| Abdominal pain | 1928 (9.4) | 151 (6.6) | 303 (6.7) | <0.001 | 1608 (8.3) | 326 (6.8) | 347 (7.4) | <0.001 | 1482 (8.1) | 725 (6.9) | 204 (7.2) | <0.001 |
| Neurological | 1322 (6.4) | 118 (5.2) | 263 (5.8) | | 1354 (7.0) | 262 (5.5) | 279 (6.0) | | 1236 (6.7) | 457 (4.4) | 159 (5.6) | |
| Uro-gynaecological | 1005 (4.9) | 70 (3.1) | 198 (4.4) | | 822 (4.2) | 177 (3.7) | 178 (3.8) | | 684 (3.7) | 473 (4.5) | 100 (3.5) | |

| | | | | | | | | | | | | |
|---|---|---|---|---|---|---|---|---|---|---|---|---|
| Fever | 596 (2.9) | 65 (2.8) | 144 (3.2) | | 519 (2.7) | 155 (3.2) | 125 (2.7) | | 548 (3.0) | 372 (3.6) | 80 (2.8) | |
| Chest pain | 532 (2.6) | 20 (0.9) | 122 (2.7) | | 466 (2.4) | 75 (1.6) | 112 (2.4) | | 464 (2.5) | 187 (1.8) | 64 (2.2) | |
| Unspecified symptoms | 11 568 (56.1) | 1354 (59.1) | 2430 (54.0) | | 10 999 (56.9) | 2835 (59.3) | 2614 (55.9) | | 10 627 (57.7) | 6228 (59.7) | 1567 (54.9) | |
| Others | 1492 (7.2) | 111 (4.8) | 348 (7.7) | | 1748 (9.0) | 268 (5.6) | 372 (7.9) | | 1628 (8.8) | 615 (5.8) | 219 (7.6) | |
| **DIAGNOSIS REGISTERED AT ED VISIT** | | | | | | | | | | | | |
| Respiratory | 2465 (12.0) | 376 (16.4) | 719 (16.0) | | 2030 (10.5) | 683 (14.3) | 679 (14.5) | | 1922 (10.4) | 1351 (12.9) | 418 (14.6) | |
| Cardiovascular | 2618 (12.7) | 199 (8.7) | 477 (10.6) | | 2561 (13.2) | 522 (10.9) | 581 (12.4) | | 2542 (13.8) | 997 (9.6) | 342 (12.0) | |
| Digestive system | 1680 (8.1) | 172 (7.5) | 323 (7.2) | | 1592 (8.2) | 368 (7.7) | 341 (7.3) | | 1544 (8.4) | 790 (7.6) | 198 (6.9) | |
| Nervous system | 966 (4.7) | 71 (3.1) | 188 (4.3) | | 1075 (5.6) | 206 (4.3) | 269 (5.8) | | 941 (5.1) | 502 (4.8) | 157 (5.5) | |
| Genito-urinary | 1422 (6.9) | 122 (5.3) | 275 (6.1) | | 1121 (5.8) | 247 (5.2) | 264 (5.6) | | 1047 (5.7) | 695 (6.7) | 146 (5.1) | |
| Neoplasm | 1071 (5.2) | 191 (8.3) | 262 (5.8) | | 767 (4.0) | 296 (6.2) | 207 (4.4) | | 588 (3.2) | 373 (3.6) | 127 (4.4) | |
| Blood diseases | 532 (2.6) | 75 (3.3) | 107 (2.4) | <0.001 | 404 (2.1) | 148 (3.1) | 81 (1.7) | <0.001 | 455 (2.5) | 360 (3.4) | 73 (2.6) | <0.001 |
| Infectious diseases | 378 (1.8) | 66 (2.9) | 104 (2.3) | | 422 (2.2) | 141 (3.0) | 72 (1.5) | | 480 (2.6) | 264 (2.5) | 63 (2.2) | |
| Endocrine | 587 (2.8) | 109 (4.8) | 123 (2.7) | | 540 (2.8) | 201 (4.2) | 136 (2.9) | | 576 (3.1) | 339 (3.2) | 87 (3.0) | |
| Osteo-muscular | 1095 (5.3) | 49 (2.1) | 147 (3.3) | | 1372 (7.1) | 154 (3.2) | 248 (5.3) | | 1274 (6.9) | 391 (3.7) | 122 (4.3) | |
| Mental health | 677 (3.3) | 58 (2.5) | 176 (3.9) | | 635 (3.3) | 124 (2.6) | 155 (3.3) | | 594 (3.2) | 304 (2.9) | 89 (3.1) | |
| Unspecified symptoms | 5167 (25.1) | 575 (25.1) | 1171 (26.0) | | 4886 (25.2) | 1213 (25.4) | 1162 (24.8) | | 4609 (25.0) | 2794 (26.8) | 716 (25.1) | |
| Access to healthcare services | 1040 (5.0) | 130 (5.7) | 229 (5.1) | | 939 (4.8) | 274 (5.7) | 256 (5.5) | | 892 (4.8) | 673 (6.4) | 157 (5.5) | |
| Others | 1305 (6.3) | 165 (7.1) | 303 (6.7) | | 1440 (7.4) | 343 (7.1) | 299 (6.3) | | 1433 (7.7) | 869 (8.3) | 222 (7.7) | |
| **TIME OF ARRIVAL AT ED** | | | | | | | | | | | | |
| 8:00AM-4:00PM | 12 119 (58.8) | 1235 (53.9) | 2474 (55.0) | | 11 325 (58.5) | 2716 (56.9) | 2656 (56.8) | | 10.720 (58.2) | 5.965 (57.1) | 1.653 (57.9) | |
| 4:00PM-12:00PM | 5041 (24.4) | 752 (32.8) | 1165 (25.9) | <0.001 | 4831 (25.0) | 1349 (28.2) | 1165 (24.9) | <0.001 | 4.661 (25.3) | 2.724 (26.1) | 720 (25.2) | 0.483 |
| 12:00PM-8:00AM | 3465 (16.8) | 305 (13.3) | 861 (19.1) | | 3206 (16.5) | 714 (14.9) | 856 (18.3) | | 3.036 (16.5) | 1.749 (16.8) | 481 (16.9) | |
| **DESTINATION AFTER DISCHARGE FROM ED** | | | | | | | | | | | | |
| Hospital | 9579 (46.4) | 1278 (55.8) | 2383 (53.0) | | 8773 (45.3) | 2285 (47.8) | 2246 (48.1) | | 8.660 (47.0) | 4.354 (41.8) | 1.384 (48.5) | |
| Home | 10 747 (52.2) | 843 (36.8) | 1777 (39.5) | <0.001 | 10 308 (53.2) | 2309 (48.3) | 2139 (45.7) | <0.001 | 9.489 (51.5) | 5.811 (55.7) | 1.273 (44.6) | <0.001 |
| Other facility | 299 (1.4) | 171 (7.4) | 340 (7.5) | | 281 (1.5) | 185 (3.9) | 292 (6.2) | | 268 (1.5) | 273 (2.5) | 197 (6.9) | |

[a] White codes (Non-urgent) are often used during IHC for planned admission; ED = Emergency Department, IHC = Integrated Home Care
